# Correlative volume-imaging using combined array tomography and FIB-SEM tomography with beam deceleration for 3D architecture visualization in tissue

**DOI:** 10.1093/jmicro/dfac015

**Published:** 2022-03-24

**Authors:** Shingo Hirashima, Keisuke Ohta, Yukiko Rikimaru-Nishi, Akinobu Togo, Takashi Funatsu, Risa Tsuneyoshi, Yuichi Shima, Kei-ichiro Nakamura

**Affiliations:** Department of Anatomy, Division of Microscopic and Developmental Anatomy, Kurume University School of Medicine, Kurume 830-0011, Japan; Dental and Oral Medical Center, Kurume University School of Medicine, Kurume 830-0011, Japan; Department of Anatomy, Division of Microscopic and Developmental Anatomy, Kurume University School of Medicine, Kurume 830-0011, Japan; Advanced Imaging Research Center, Kurume University School of Medicine, Kurume 830-0011, Japan; Department of Anatomy, Division of Microscopic and Developmental Anatomy, Kurume University School of Medicine, Kurume 830-0011, Japan; Department of Plastic and Reconstructive Surgery and Maxillofacial Surgery, Kurume University School of Medicine, Kurume 830-0011, Japan; Advanced Imaging Research Center, Kurume University School of Medicine, Kurume 830-0011, Japan; Advanced Imaging Research Center, Kurume University School of Medicine, Kurume 830-0011, Japan; Department of Anatomy, Division of Microscopic and Developmental Anatomy, Kurume University School of Medicine, Kurume 830-0011, Japan; Department of Anatomy, Division of Microscopic and Developmental Anatomy, Kurume University School of Medicine, Kurume 830-0011, Japan; Department of Anatomy, Division of Microscopic and Developmental Anatomy, Kurume University School of Medicine, Kurume 830-0011, Japan; Cognitive and Molecular Research Institute of Brain Diseases, Kurume University School of Medicine, Kurume 830-0011, Japan

**Keywords:** mesoscopic architecture, volume imaging, three-dimensional analysis, FIB-SEM, array tomography, serial block-face scanning electron microscopy

## Abstract

Focused ion beamed (FIB) SEM has a higher spatial resolution than other volume-imaging methods owing to the use of ion beams. However, in this method, it is challenging to analyse entire biological structures buried deep in the resin block. We developed a novel volume-imaging method by combining array tomography and FIB-SEM tomography and investigated the chondrocyte ultrastructure. Our method imparts certainty in determining the analysis area such that cracks or areas with poor staining within the block are avoided. The chondrocyte surface showed fine dendritic processes that were thinner than ultrathin sections. Upon combination with immunostaining, this method holds promise for analysing mesoscopic architectures.

Various volume-imaging methods, such as array tomography (AT), automatic tape-collecting ultramicrotome (ATUM), serial block-face (SBF-SEM), focused ion beamed (FIB) SEM, have been developed to date [1‒5]. Analyses on the applications of volume imaging and correlative light and electron microscopy (CLEM) have also been performed for three-dimensional (3D) ultrastructural analysis in various research fields [2,6]. Each of these methods has advantages as well as disadvantages. Our previous studies have presented the results of the analysis of biological ultrastructures using FIB-SEM, which has the ability to identify the areas to be analysed through extensive block-face imaging (BFI) of complex tissues [7,8]. FIB-SEM has a higher spatial resolution than other volume-imaging methods owing to the use of ion beams. Using beam deceleration (BD) along with FIB-SEM is advantageous in BFI of biological specimens embedded in resin under low-voltage and low-beam current conditions because BD improves image contrast and resolution [9]. However, as the FIB-SEM observation method is a ‘slice and view’ method that needs to be performed repetitively and automatically using a focused ion beam and SEM, the observed area is not preserved and the same area cannot be observed again. Furthermore, although the observation site is identified by BFI, analysing the structure of entire cells and other structures buried deep in the block is not occasionally feasible. These are the limitations associated with FIB-SEM. SBF-SEM is similar in this context because the observation area cannot be preserved with this method either. On the other hand, as numerous serial ultrathin sections placed on glass slides or silicon wafers (multiple columns of ribbon) are preserved in AT and ATUM, the sections can be observed repetitively. However, preparing hundreds or thousands of serial ultrathin sections without error is extremely difficult. The length Z in the voxel size of AT or ATUM refers to the thickness of an ultrathin section, which is usually 60–80 nm. If a biological ultrastructure has a thickness lesser than this value, it will be buried in the ultrathin section. Therefore, there is a need for a simpler, higher-resolution analysis that is more efficient and requires fewer tasks.

In this study, we developed a novel volume-imaging method that combines AT and FIB-SEM tomography with BD and utilizes the merits of both techniques (Fig. 1). Furthermore, we investigated the 3D architecture of hyaline cartilage in tissues using this novel volume-imaging method.

**Fig. 1.**
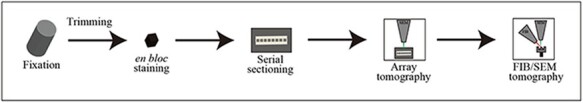
Scheme of correlative volume-imaging combined array tomography and FIB-SEM tomography. First, a fixed specimen was trimmed and then *en bloc* stained. Serial sections were cut into ribbons and mounted on a silicon wafer. Second, serial sections were imaged using array tomography. After observation of the stack images and the 3D images, the ROI for FIB-SEM tomography was identified. The silicon wafer mounted on serial semithin sections was cut and placed on a customized holder. The ROI area was determined and observed using FIB-SEM.

All experiments were performed in accordance with the National Institutes of Health guidelines for animal research. All animal procedures were approved by the Board for Animal Experiments at the Kurume University.

Specimens were prepared as described in our previous studies [9‒14]. Mice (C57BL/6, 4 weeks old) were anesthetized using a mixture of three anaesthetics, namely medetomidine hydrochloride, midazolam and butorphanol [15,16]. The anaesthetic mixture was prepared by mixing 0.75 mL of medetomidine hydrochloride, 2 mL of midazolam and 2.5 mL of butorphanol, and the volume was made up to 25 mL with saline solution. This anaesthetic combination was prepared to achieve a dosage of 0.3 mg/kg medetomidine hydrochloride, 4.0 mg/kg midazolam and 5.0 mg/kg butorphanol. Then, mice were transcardially perfused through the left ventricle with heparin (10 U/mL) in saline, followed by fixation with 2% paraformaldehyde and incubation in 2.5% glutaraldehyde in 0.1 M cacodylate buffer (pH 7.3). After perfusion, the rib cartilage (hyaline cartilage) was dissected from the thoracic cage. The specimens were immersed in the same fixative for 2 h at 4°C and washed in 0.1 M cacodylate buffer, after which the specimens were washed three times in buffer for 10 min each.

Briefly, after three washes in cacodylate buffer, the specimens were post-fixed for 2 h in a solution containing 2% osmium tetroxide and 1.5% potassium ferrocyanide in cacodylate buffer at 4°C, washed three times with distilled water (DW) and immersed in 1% thiocarbohydrazide solution for 1 h. After five washes with DW, the specimens were immersed again in 2% osmium tetroxide in DW and then washed three times with DW. The specimens were then stained *en bloc* in a solution of 4% uranyl acetate dissolved in DW overnight for contrast enhancement and then washed with DW. Subsequently, the specimens were further stained with Walton’s lead aspartate solution for 2 h, dehydrated in an ethanol series (25%, 50%, 70%, 80% and 90% and twice in 100% ethanol for 5 min each), infiltrated with epoxy resin (Epon 812; TAAB, Aldermaston, UK) and polymerized for 72 h at 60°C.

The resin block with the embedded specimens was trimmed using an EM TRIM2 trimmer (Leica, Wetzlar, Germany). The surfaces of the embedded specimens were exposed. After trimming, ribbons of serial semithin sections were cut with an ultramicrotome ARTOS 3D (Leica) and a histo jumbo diamond knife (Diatome; Nisshin EM, Tokyo, Japan). The thickness of the sections was 1 µm. To cut ribbons of serial sections, Bond G17 adhesive (Konishi, Osaka, Japan) was diluted in xylene and applied with a needle to the bottom of the block pyramid [2,17]. After the adhesive had dried for 2 min at room temperature (26°C), a series of 10 or more sections was cut. The ribbons were mounted on silicon wafers. To prevent charging, the ribbons were coated with a thin layer of evaporated osmium (Neoc-Pro, Meiwafosis, Tokyo, Japan).

For AT, an outline image of entire ribbons imaged with MVX10 (Olympus, Tokyo, Japan), Digital sight1000 (Nikon, Tokyo, Japan) (Fig. 2a), JSM-IT8000 (JEOL, Tokyo, Japan) and the SM-Z19068TRAYS (JEOL) software was used (Fig. 2b). The settings for SEM observation were fixed as follows: landing energy = 7.0 KeV, working distance = 9.0 mm, dwell time = 14.6 µs/pixel, image size = 2560 × 1920 pixels, colour depth = 8 bit (256 grey scales), pixel size = 16.6 nm/pixel and beam current = 23 pA. Images were obtained using a solid-state backscattered electron detector. The stack images obtained were exported to Fiji (http://fiji.sc/Fiji) and Amira 2020.2 (FEI Visualization Science Group, Burlington, MA, USA).

**Fig. 2.**
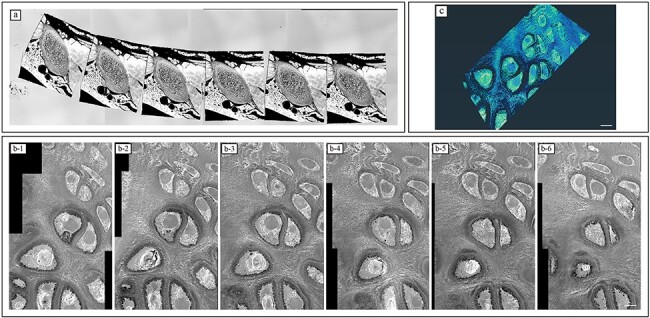
Array tomography. (a) Outline image of entire ribbons. (b) Stack image of the ROI. (c) 3D images of the ROI using volume rendering. After extensive block-face imaging, the area for FIB-SEM tomography was selected. Scale bar = 5 µm.

After observation of the stack images and the 3D images, the region of interest (ROI) for FIB-SEM tomography was identified (Fig. 2b and c). The silicon wafer mounted on the serial semithin sections was cut to dimensions 4 × 25 mm. Then, the silicon wafer was placed on a modified version of the customized holder (Fig. 3) using conductive carbon double-coated tape, which we reported previously [9], to maintain a horizontal attitude and to minimize the effect of specimen tilt during ion beam milling and image acquisition cycles under the cathode bias field. FIB-SEM tomography analysis was performed as previously described [9,10]. Serial images of the ROI area were acquired via repeated cycles of sample surface milling and imaging using the Slice & View G2 operating software (FEI, Eindhoven, Netherlands). Milling was performed with a gallium ion beam at 30 kV with a current of 1.0 nA. The milling pitch was set at 10 nm/step and 800 cycles. Images were acquired at a landing energy of 2.0 keV and the BD was 2.5 KeV. Additional acquisition parameters were as follows: beam current = 24.9 pA, dwell time = 6 µs/pixel, image size = 2048 × 1768 pixels and pixel size = 2.9 nm/pixel or 3.6 nm/pixel (voxel size = 2.9 × 2.9 ×10 nm or 3.6 × 3.6 × 10 nm). The magnification was ×25 000. The images were obtained using a solid-state backscattered electron detector (VCD; FEI). The resulting image stack, segmentation and 3D-reconstructed images were processed using the Amira 2020.2 software (FEI Visualization Science Group, Burlington, MA, USA). The 3D morphology of the cells was reconstructed using volume rendering and manual procedure.

**Fig. 3.**
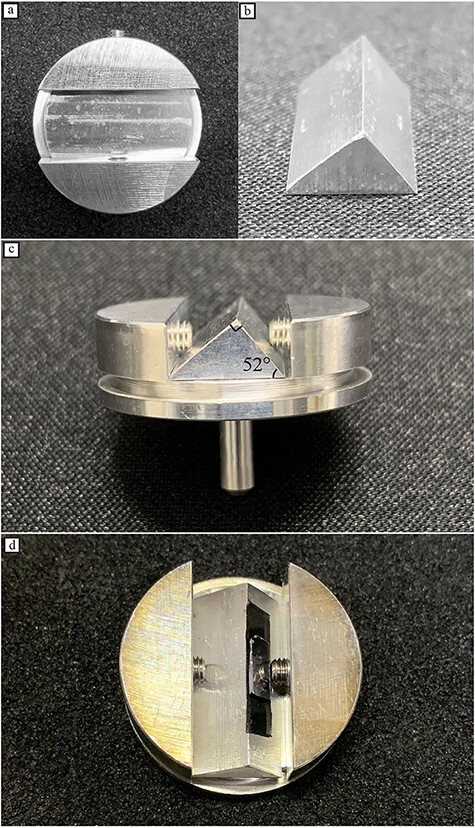
Modified sample holder for FIB-SEM observation with cathode bias condition. (a) Groove to set sample table, (b) sample table, (c) customized holder and (d) serial sections and silicon wafer attached to the customized holder. One angle of the sample table was set at 52 to maintain the horizontal attitude of the holder and to minimize the effect of specimen tilt during ion beam milling and image acquisition cycles under the cathode bias field. The serial sections on the silico wafer were attached to the customized holder using conductive carbon double-coated tape.

Fine dendritic processes were observed in the serial cross-sections (Fig. 4). Due to the thickness of the semithin section, the surface of the ribbon (** in Fig. 4) was visible. Fibrous structures were observed in the interstitial cartilage (*** in Fig. 4). In the 3D reconstruction images, the surface of the chondrocytes had many fine dendritic processes, which were not smooth and flat. The processes were club-shaped and approximately 300 nm in length and 30 nm in width (Fig. 5).

**Fig. 4.**
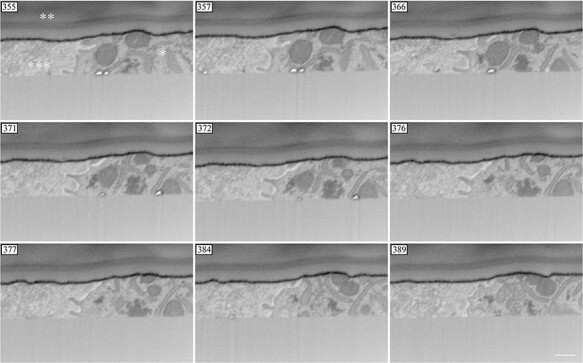
FIB-SEM tomography: serial cross-section. Fine dendritic processes of the chondrocytes were observed.

*: cell body of chondrocytes, **: the surface of ribbons, ***: interstitium of hyaline cartilage. Scale bar = 500 nm.

**Fig. 5.**
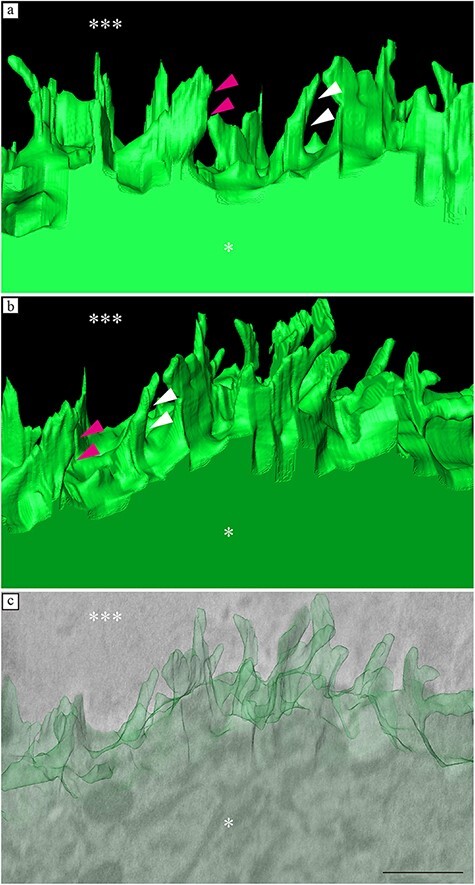
FIB-SEM tomography: 3D reconstruction. Surface of the chondrocytes, showing many fine club-shaped or flat-shaped dendritic processes, which were not smooth and flat (white and magenta arrowheads).

*: cell body of chondrocytes, ***: interstitium of hyaline cartilage. Scale bar = 500 nm.

In the present study, we combined AT and FIB-SEM to develop a novel 3D mesoscopic analysis method. This method allowed for the imaging of the 3D ultrastructure of chondrocytes that remain hidden due to the thickness of conventional ultrathin sections. In light microscopy (LM), chondrocytes are characterized as round cells [18], whereas in conventional electron microscopy (EM), they are characterized as cells with protrusions [19].

Transmission electron microscopy (TEM) observations have shown that the surface of chondrocytes have long and prominent cytoplasmic processes [19]. However, in conventional TEM analysis (2D observation), it is difficult to visualize the 3D morphology of these cellular processes. Whether plate-like or club-shaped in 3D, a structure appears as the same dendritic process in 2D analysis. In the present study, we showed that the surface of chondrocytes had multiple and very fine club-shaped cellular processes. The thickness of a club-shaped process was approximately 30 nm, which is difficult to observe in conventional ultrathin sections. The shape of the chondrocytes in a healthy cartilage is a smooth ellipse or spheroid, when observed through LM [20]. Based on these reports and our results, it is suggested that chondrocytes are elliptical or spheroid-shaped cells with fine club-shaped dendritic processes on their surface and not simply smooth ellipses or spheroid-shaped structures.

ATUM-FIB microscopy, which utilizes the merits of ATUM and FIB-SEM, has been reported previously [21,22]. This innovative method is beneficial in the morphological analysis of biological events. The ATUM method requires a tape collector, which needs to be adjusted to collect serial ultrathin sections. Therefore, AT is considered to be a simpler method than ATUM. In light of this, we developed a simpler method that can be used to analyse mesoscale architecture with a high resolution using AT. Our novel method can also be used for 3D mesoscopic analysis at scales of tens of nanometres.

We performed *en bloc* staining, which is frequently used for FIB-SEM analysis. Various analytical methods combining AT and multiple immunostaining methods have been reported [2]. Our novel volume-imaging method has numerous potential applications because the following steps in the process of *en bloc* staining can be adjusted: length of staining time [23] and use of additional tannic acid. Furthermore, the combination with CLEM can expand the range of applications of image analysis. In future studies, we intend to adjust the staining method according to the structure to be analysed, combine this method with immunostaining and apply it to the analysis of mesoscopic architectures smaller than ultrathin sections.

Because AT was performed on semithin sections in our method, the samples were prone to knife marks. Sample preparation at this thickness with a diamond knife is arduous, and the condition of the diamond knife requires more attention than that in conventional methods. Furthermore, as the semithin section was 1 µm, uneven thicknesses, cracks and poorly stained areas were often observed in serial sections (Fig. 2b). Contrarily, in conventional FIB-SEM tomography with a block sample, even when no abnormality exists on the block surface, cracks or areas with poor staining within the block are unavoidable during data acquisition. In contrast, our method can detect areas with poor staining or cracks, which makes it easier to avoid such areas during FIB-SEM tomography.

Our novel method has a couple of limitations. First, the size of the groove of the customized holder was limited (4 × 25 mm), and this necessitated trimming of the silicon wafer after AT imaging. As there is a possibility of sample damage during trimming, careful trimming is necessary when selecting the area to be imaged using FIB-SEM. Second, because the ribbon of serial sections attached on the silicon wafers is trimmed and its continuity is lost or disrupted, repeated observation of intact serial sections is difficult, as in conventional AT.

In this study, we developed a novel volume-imaging method that combines array tomography and FIB-SEM tomography with beam deceleration. AT is a simple volume-imaging technique that can be performed using SEM and serial sections. Our novel method enabled imaging with a higher resolution and imparted more certainty in determining the analysis area for FIB-SEM.
